# Predictors of distant metastasis on exploration in patients with potentially resectable pancreatic cancer

**DOI:** 10.1186/s12876-018-0891-y

**Published:** 2018-11-06

**Authors:** Xinchun Liu, Yue Fu, Qiuyang Chen, Junli Wu, Wentao Gao, Kuirong Jiang, Yi Miao, Jishu Wei

**Affiliations:** 1 Pancreas Center, The First Affiliated Hospital of Nanjing Medical University, 300 Guangzhou Road, Nanjing, Jiangsu Province, China; 2 Pancreas Institute, Nanjing Medical University, Nanjing, China; 3 Department of Gastrointestinal Surgery, The Affiliated Changzhou No.2 People’s Hospital of Nanjing Medical University, Changzhou, China

**Keywords:** Distant metastasis, Pancreatic cancer, Predictive factor, Surgical exploration

## Abstract

**Background:**

Patients with potentially resectable pancreatic ductal adenocarcinoma (PDAC) are frequently found to be unresectable on exploration due to small distant metastasis. This study was to investigate predictors of small distant metastasis in patients with potentially resectable PDAC.

**Methods:**

Patients who underwent surgical exploration for potentially resectable PDAC from 2013 to 2014 were reviewed retrospectively and divided into two groups according to whether distant metastases were encountered on exploration. Then, univariate and multivariate logistic regression analyses were used to identify predictors of distant metastasis. A scoring system to predict distant metastasis of PDAC on exploration was constructed based on the regression coefficient of a multivariate logistic regression model.

**Results:**

A total of 235 patients were included in this study. Mean age of the study population was 61.7 ± 10.4 years old. Upon exploration, distant metastases were found intraoperatively in 62 (26.4%) patients, while the remaining 173 were free of distant metastases. Multivariate logistic regression analysis identified that age ≤ 62 years old (*p* < 0.001), male sex (*p* = 0.011), tumor size ≥4.0 cm (*p* < 0.001), alanine aminotransferase level (ALT) < 125 U/L (*p* < 0.001), and carbohydrate antigen (CA19–9) level ≥ 385 U/mL (*p* < 0.001) were independent risk factors for occult distant metastasis of PDAC. A preoperative scoring system (0–8 points) for distant metastasis on exploration was constructed using these five factors. The receiver operating characteristic curves showed that the area under the curve of this score was 0.85. A score of 6 points was suggested to be the optimal cut-off value, and the sensitivity and specificity were 85% and 69%, respectively.

**Conclusions:**

Distant metastasis is still frequently encountered on exploration for patients with potentially resectable PDAC. Younger age, male sex, larger tumor size, low ALT level and high CA19–9 level are independent predictors of unexpected distant metastasis on exploration.

## Background

Pancreatic ductal adenocarcinoma (PDAC) is one of the most dismal malignancies with an overall 5-year survival rate of < 7% [1, 2]. Despite enormous efforts directed at the treatment of PDAC, radical resection remains the most effective treatment modality, and it increases the 5-year survival rate for PDAC patients to 10–25% [3–5]. However, due to a lack of presentations at early stages and the aggressive nature of this disease, the majority of PDAC patients present an unresectable disease at the time of diagnosis, and only around 20% of newly diagnosed PDAC patients were suitable candidates for curable surgical resection [6].

Multidetector computed tomography (MDCT) is currently the optimal imaging modality for preoperative diagnosis and staging of PDAC [7, 8]. However, this imaging modality has a poor sensitivity for identifying small liver or peritoneal metastasis [7, 9]. Among the patients subjected to surgical exploration, a significant proportion (40%) of them are found to be unresectable due to occult distant metastasis or infiltration of local structures [10–12]. The proportion of patients successfully resected during surgical exploration might be as low as 50% [12, 13].

For patients with distant occult metastasis, surgical resection is unnecessary as it does not prolong survival in the overwhelming majority of patients [14, 15]. Besides, unnecessary surgical exploration often delays administration of other treatments, for example systematic chemotherapy, which currently is the preferred treatment for metastatic PDAC patients [16]. Therefore, it is important to differentiate PDAC patients with distant metastasis from those with truly resectable cancers to avoid unnecessary surgery and offer these patients tailored treatments in a timely manners. The objective of this retrospective study was to analyze the predictive factors for distant occult metastasis in patients with resectable PDAC based on preoperative MDCT.

## Methods

### Study design and patients

This was a single institution, retrospective study, from a high-volume center, the Pancreas Center, The First Affiliated Hospital of Nanjing Medical University, China. All patients who underwent elective pancreatic surgery at our unit between January 2014 and December 2015 were reviewed retrospectively. Only patients with a final diagnosis of PDAC were included. Exclusion criteria were as follows: 1) patients underwent an operation with palliative intent, 2) patients without preoperative internal MDCT, and 3) patients with distant metastasis detected with preoperative MDCT. All patients underwent a triple-phase 16-row MDCT, consisting of unenhanced, early arterial, and venous phases.

Patients were included in the “with metastasis” (WM) group when distant metastasis, such as liver and peritoneal metastasis, was encountered during surgery. The remaining patients were included in the “no metastasis” (NM) group. During the surgery, distant metastasis was discovered through manual palpation by experienced surgeons and further confirmed with frozen resection. Intra-operative ultrasound was not used.

Data collected included age at diagnosis; sex; drinking and smoking history; comorbidities (Hypertension and Diabetes Mellitus); chief complaint (with pain or without pain); preoperative laboratory data, such as alanine aminotransferase (ALT), aspartate aminotransferase (AST), total bilirubin (TBil), direct bilirubin (DBil), albumin, alpha fetoprotein (AFP), carbohydrate antigen (CA19–9), and carcinoembryonic antigen (CEA); tumor size and location on MDCT; and time interval between MDCT and operation. The possible risk factors for distant metastasis were then examined statistically. Data were obtained from the patients’ medical records and the hospital electronic database. All the imaging results were reviewed by a dedicated radiologist. This study was approved by the institutional review board with a waiver of informed consent (No. 2016-SR-210).

### Statistical analysis

Quantitative variables are presented as the mean ± standard deviation and qualitative variables are expressed as absolute and relative frequencies. Comparisons between the WM and NM groups are performed using the Student’s *t*-test or Chi-square test accordingly. The association between the predictive factors and presence of distant metastasis was first evaluated by univariate logistic regression. Factors with a *p* < 0.1 in the univariate regression analysis were included in multivariate logistic regression analysis. Backward stepwise elimination was used to exclude variables with *p* > 0.05 from the model. Continuous variables were divided into two groups according to the mean value of each parameter. All statistical analyses were performed using Stata/SE version 10.0 for Windows (StataCorp, Texas, USA). All tests for significance were two-sided and a value of *p* < 0.05 was considered statistically significant.

## Results

### Demographic and clinicopathologic characteristics

**Fig. 1:**
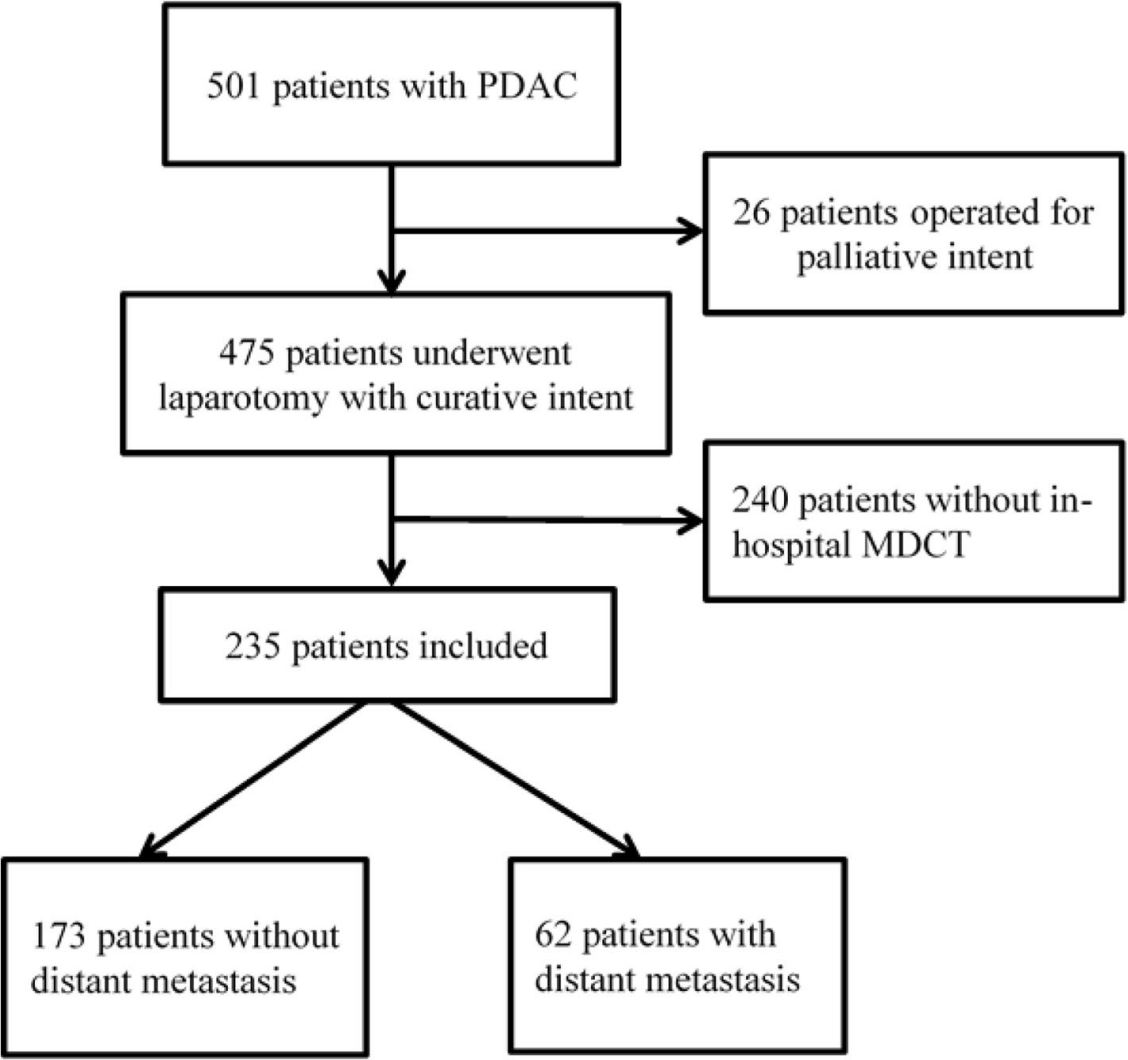
Flow diagram showing selection of patients for inclusion in the study

In the study period, a consecutive series of 501 patients with PDAC underwent laparotomy in our center. Of these, 26 patients were excluded because they had unresectable disease detected radiologically and underwent an operation with palliative intent. Another 240 patients were excluded for having no internal MDCT: 218 didn’t have any image studies in our hospital, and 22 had only Magnetic Resonance Imaging or magnetic resonance cholangiopancreatography or positron emission tomography/computed tomography other than MDCT. Ultimately, a total of 235 patients were included in the analysis (Fig. 1).

All the included 235 patients underwent upfront surgery, and received no neoadjuvant therapy. Distant metastasis was found in 62 (26.4%) patients, including 31 liver metastases and 31 peritoneal metastases. Of the 62 patients with metastases, three patients underwent pancreaticoduodenectomy for primary cancer and the remaining patients underwent different palliative procedures accordingly. Of the 173 NM patients, 164 patients underwent resection successfully and 9 patients underwent palliative operations because the tumor was locally advanced. Details of the procedures are shown in Table 1.

**Table 1:** Procedures performed for 235 patients

	Total (*n* = 235)	No Metastasis (*n* = 173)	With Metastasis (*n* = 62)
Resected	167	164	3
PD/PPPD	124	121	3
Distal pancreatectomy	39	39	0
Total pancreatectomy	1	1	0
Appleby Operation	3	3	0
Not Resected	68	9	59
Double Bypass	13	1	12
Biliary Bypass	9	4	5
Gastric Bypass	4	0	4
Celiac plexus neurolysis	16	0	16
Exploration alone	26	4	22

*PD* pancreaticoduodenectomy, *PPPD* pylorus-preserving pancreaticoduodenectomy

### Comparisons between the patients with metastasis (WM) and patients with no metastasis (NM)

Patients’ demographics and laboratory values at the time of diagnosis are shown in Table 2. The mean age of all patients was 61.7 ± 10.4 years (median 62 years, range 29–87 years), and 64.3% (*n* = 151) were male. The metastasis group had a younger age (59.4 vs. 62.5 years, *p* = 0.041) and larger tumor size compared with the NM group (4.2 vs. 3.8 cm, *p* < 0.001). Additionally, patients in the NM group had a higher ALT level (*p* = 0.010) and a higher AST level (*p* = 0.010) when compared with patients in the WM group. Levels of TBil and DBil in the WM group were found to be lower than those in the NM group; however, these differences were not significant (*p* = 0.057 and 0.085, respectively).

**Table 2:** Demographic and clinical characteristics of included patients

	Total (*n* = 235)	NM (*n* = 173)	WM (*n* = 62)	*p*
Age (years) (mean ± SD)	61.7 ± 10.4	62.5 ± 10.4	59.4 ± 10.0	0.041
Sex, male/female	151/84	105/68	46/16	0.057
Chief complaint				
Pain	150	106	44	0.173
Without pain	85	67	18	
Jaundice	73	61	12	0.200
Without jaundice	162	112	50	
Weight loss	100	70	30	0.279
Without weight loss	135	103	32	
Personal history				
Smoking, yes/no	48/187	34/139	14/48	0.624
Drinking, yes/no	38/197	32/141	6/56	0.106
Hypertension, yes/no	71/164	51/122	20/42	0.683
Diabetes, yes/no	36/199	25/148	11/51	0.537
Interval between imaging and surgery, days	6.3 ± 4.8	6.3 ± 4.6	6.2 ± 5.2	0.868
Tumor size on MDCT (cm)	4.2 ± 1.9	3.8 ± 1.6	5.5 ± 2.0	< 0.001
Tumor location				
Head	174	134	40	0.046
Body or tail	61	39	22	
Laboratory examinations				
ALT	124.6 ± 173.1	142.2 ± 179.1	75.9 ± 146.1	0.010
AST	84.3 ± 100.5	94.4 ± 105.0	56.3 ± 81.2	0.010
TBil	73.9 ± 103.8	81.6 ± 107.6	52.4 ± 89.5	0.057
DBil	50.3 ± 74.6	55.4 ± 77.2	36.3 ± 65.7	0.085
ALB	40.0 ± 5.4	40.1 ± 5.4	39.7 ± 5.2	0.686
AFP	2.9 ± 1.8	2.8 ± 1.4	3.1 ± 2.6	0.293
CA19–9	385.5 ± 378.8	335.7 ± 350.2	525.2 ± 422.0	< 0.001
CEA	8.9 ± 20.3	8.1 ± 21.9	11.1 ± 14.7	0.328

### Predictive factors for occult distant metastases on exploration

Table 3 summarizes the univariate and multivariate logistic regression analyses of the risk factor for distant metastasis using the significant univariate predictors. In univariate analyses, significant predictive factors for finding distant metastasis during surgery were younger age (*p* = 0.003), larger tumor size (*p* < 0.001), tumor location (*p* = 0.048), lower ALT level (*p* < 0.001), lower AST level (*p* = 0.006), lower TBil level (*p* < 0.019), higher CA199 level (*p* = 0.007), and higher CEA level (*p* < 0.001) (Table 3). In multivariate analysis, the following variables remained significantly associated with presence of distant metastasis: an age < 62 years old (Odds ratio (OR) = 3.97; 95% Confidence interval (CI): 1.87–8.42; *p* < 0.001), male sex (OR = 2.79, 95% CI: 1.26–6.19; *p* = 0.011), a tumor size ≥4.0 cm (OR = 16.02, 95% CI: 5.31–48.30; *p* < 0.001), ALT level < 125 U/L (OR = 6.19, 95% CI: 2.26–16.92, *p* < 0.001), and a CA19–9 level ≥ 385 U/ml (OR = 3.53, 95% CI: 1.87–6.67; *p* < 0.001) (Table 3).

**Table 3:** Univariate and multivariate analyses of factors predicting distant metastases

		Total *n* = 235	NM *n* = 173	WM *n* = 62	Univariate analysis		Multivariate analysis^a^	
					*p*	OR (95% CI)	*p*	OR (95% CI)
Age	> 62	115	95	20	0.003	1	< 0.001	1
	≤62	120	78	42		2.55 (1.39, 4.71)		3.97 (1.87, 8.42)
Sex	Female	84	68	16	0.057	1	0.011	1
	Male	151	105	46		1.86 (0.98, 3.55)		2.79 (1.26, 6.19)
Pain	No	85	67	18	0.174	1		
	Yes	150	106	44		1.50 (0.82, 2.90)		
Jaundice	No	162	112	50	0.022	1		
	Yes	73	61	12		0.44 (0.22, 0.89)		
Weight loss	No	135	103	32	0.280	1		
	Yes	100	70	30		1.37 (0.77, 2.47)		
Smoking	No	187	139	48	0.624	1		
	Yes	48	34	14		1.19 (0.59, 2.41)		
Drinking	No	197	141	56	0.112	1		
	Yes	38	32	6		0.47 (0.19, 1.19)		
Hypertension	No	71	51	20	0.683	1		
	Yes	164	122	42		1.14 (0.61, 2.13)		
Diabetes	No	199	148	51	0.538	1		
	Yes	36	25	11		1.28 (0.59, 2.78)		
Interval between imaging and surgery	≤7	170	123	47		1		
	7–14	48	39	9	0.213	0.60 (0.27, 1.34)		
	≥14	17	11	6	0.461	1.49 (0.52, 4.31)		
Tumor size	< 4.0	96	92	4	< 0.001	1	< 0.001	1
	≥4.0	139	81	58		16.47 (5.73, 47.36)		16.02 (5.31, 48.30)
Tumor location	Head	174	134	40	0.048	1		
	Body/tail	61	39	22		1.88 (1.01, 3.55)		
ALT	≥125	71	64	7	< 0.001	1	< 0.001	1
	< 125	164	109	55		4.61 (1.98, 10.74)		6.19 (2.26, 16.92)
AST	≥ 85	75	64	11	0.006	1		
	< 85	160	109	51		2.72 (1.32, 5.60)		
TBil	≥75	78	65	13	0.019	1		
	< 75	157	108	49		2.27 (1.14, 4.50)		
DBil	≥50	75	61	14	0.069	1		
	< 50	160	112	48		1.87 (0.95, 3.66)		
ALB	≥40	124	89	35	0.498	1		
	< 40	111	84	27		0.82 (0.46, 1.47)		
AFP	< 3.0	153	111	42	0.612	1		
	≥3.0	82	62	20		0.85 (0.46, 1.57)		
CA19–9	< 385	144	115	29	0.007	1	0.015	1
	≥385	91	58	33		2.26 (1.25, 4.07)		2.49 (1.19, 5.21)
CEA	< 9	183	145	38	< 0.001	1		
	≥9	52	28	24		3.53 (1.87, 6.67)		

*NM*, No metastases; *WM*, with metastases

^a^A multivariable model was constructed by a backward stepwise method

**Fig. 2:**
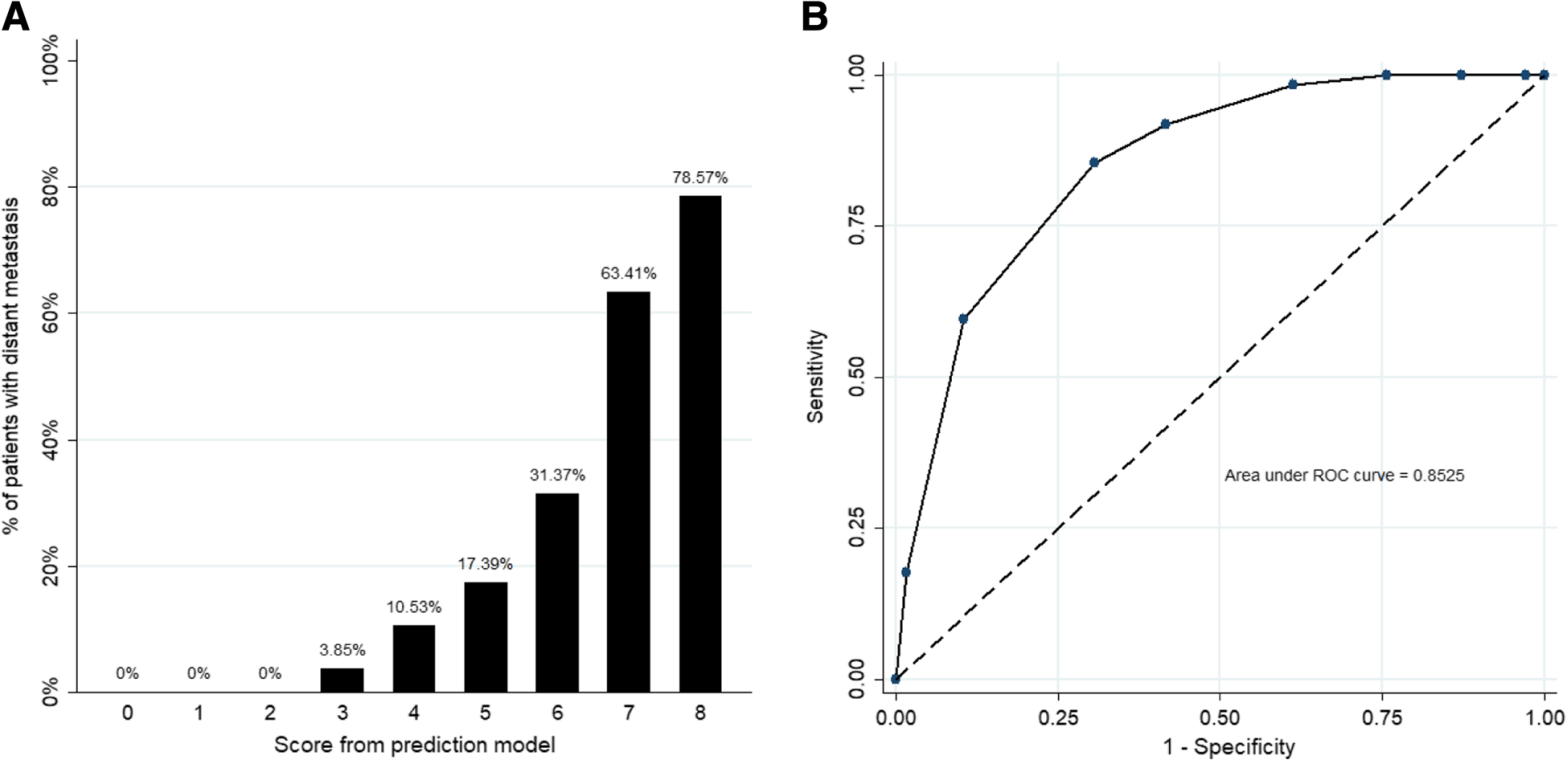
Prediction of distant metastasis. **a** Proportion of patients with occult metastasis during laparotomy. **b** Receiver operating characteristics of number of factors to predict the risk for distant metastasis found at operation

The five independent risk factors found in the multivariate analysis were used to develop a score system based on the regression coefficient of the multivariate logistic regression model (Table 4). The score values for individual patient ranged from 0 to 8. The risk of patients with distant metastasis progressively increased as the score increased (Table 5, Fig. 2a). A receiver operating characteristic curve of the model showed that the area under curve of this score was 0.85 (95% CI: 0.80–0.89) (Fig. 2b). A score of 6 points was suggested to be the optimal cut-off value (Youden index = 0.548) to divide the risk strata with a sensitivity of 85% and a specificity of 69%.

**Table 4:** Predictive scoring system for pancreatic fistula

Preoperative factor	β coefficient	Points contributed
Age		
> 62 years old		0 point
≤ 62 years old	1.38	1 point
Sex		
Female		0 point
Male	1.02	1 point
Tumor size		
< 4.0 cm		0 point
≥ 4.0 cm	2.78	3 points
ALT		
≥ 125 U/L		0 point
< 125 U/L	1.89	2 points
CA19–9		
< 385 U/mL		0 point
≥ 385 U/mL	0.91	1 point

**Table 5:** Risk of distant metastasis for patients with each score

Score	No. of patients		%				
	Total	WM		Sensitivity (%)	Specificity (%)	Accuracy (%)	Youden Index (%)
0	5	0	0	100	0	26.38	0
1	17	0	0	100	2.89	28.51	2.89
2	20	0	0	100	12.72	35.74	12.72
3	26	1	3.85	100	24.28	44.26	24.28
4	38	4	10.53	98.39	38.73	54.47	37.12
5	23	4	17.39	91.94	58.38	67.23	50.32
6	51	16	31.37	85.48	69.36	73.62	54.84
7	41	26	63.41	59.68	89.60	81.70	49.28
8	14	11	78.57	17.74	98.27	77.02	16.01

## Discussion

Currently, radical resection provides the only chance for long-term survival for patients with PDAC. As surgical skills and perioperative management developed, mortality after pancreatic surgery has dramatically decreased to less than 5% [17]. However, morbidity after pancreatic surgery is still very high. Non-curative exploratory laparotomy of pancreas can have a morbidity as high as 42.3% and does not increase survival [18]. Moreover, this unnecessary operation can postpone other more suitable therapies such as chemotherapy and can become the last straw to their debilitating state.

Unfortunately, not all patients with PDAC who undergo resection surgery can be resected successfully. Despite thorough pre-operative staging with advanced imaging techniques, incidental occult distant metastasis from PDAC is commonly encountered in during surgery [19]. Previous studies revealed that up to 31% of patients with resectable PDAC staged by MDCT were found to have metastases in sbusequent laparotomy or staging laparoscopy [8, 20–22]. In patients with locally advanced PDAC, the likelihood of finding unresectable PDAC at operation is much higher [23].

Despite the emerging use of magnetic resonance imaging, endoscopic ultrasound, and positron emission tomography/computed tomography, MDCT remains the most commonly used imaging modality for the diagnosis and staging of PDAC [7, 24, 25]. However, small distant metastases, such as minimal peritoneal deposits and small liver metastases, can remain undetected even with modern computed tomography protocols [26]. Previous studies suggested that patients with PDAC should undergo the operation within 25 or 32 days of diagnostic imaging to reduce the risk of tumor progression to unresectable disease [27, 28]. In the present study, we found that 26% of the patients selected for curative surgery for PDAC had distant metastasis. However, in our study, we found no affects attributable to the time interval between MDCT study and surgery on the accuracy of MDCT in determining the presence or absence of metastatic disease.

Due to the limitation of imaging, other techniques were reported in literature for determining the resectability of PDAC. One such technique is peritoneal lavage cytology (PLC), which is a routinely applied in the diagnosis and staging of several cancers. However, in PDAC, although a positive PLC represents an early recurrence and a worse prognosis, a positive PLC is not regarded as equal to a macrometastasis in patients with PDAC and it does not exclude a curative resection in patients without other distant metastasis [29–31]. Another technique is staging laparoscopy, which has been used to diagnose occult metastasis to decrease the number of unnecessary laparotomies in PDAC [32–34]. Patients who were found to harbor distant metastasis by laparoscopy staging received palliative chemotherapy earlier and lived longer than patients who underwent only laparotomy [33]. Moreover, a cost analysis indicated that use of laparoscopy in pancreatic cancer did not significantly increase the overall expense of treatment [34]. A recent review of 1146 patients found that diagnostic laparoscopy prior to laparotomy could decrease the rate of unnecessary laparotomy from 40 to 20% in patients with periampullary cancer [10]. As a minimally invasive modality, staging laparoscopy was suggested to be routinely used to identify radiographically occult metastases and prevent rewardless laparotomies [20, 21, 35, 36]. However, as the proportion of patients found to have metastases at laparoscopy is decreasing, its routine use is challenged, and some studies have investigated the indications for selective use of staging laparoscopy in pancreatic cancer [37]. Identifying patients at an increased risk of distant metastasis seems to be a more reasonable approach, that can increase the diagnostic accuracy of staging laparoscopy and deliver optimal disease management.

By comparing a number of preoperative factors, this study identified that young age, male sex, low ALT level, large tumor size, and high CA 19–9 level were independent predictors of distant metastases in patients with resectable PDAC. Previous studies found that tumors in the pancreas body and tail, tumor size as determined by MDCT, serum CA 19–9 level, CEA, and weight loss were risk factors for unresectability in patients with potentially resectable PDAC [20, 38–41]. Our study confirmed that tumor in the body and tail, and high CEA were associated with distant metastasis in univariate analysis, but not in multivariate analysis. Weight loss was not associated with distant metastasis. In line with previous studies, CA19–9 and tumor size were independent predictive factors for distant metastasis [37]. Ong et al. found that age < =65 was a predictive factor of resectable disease [42]. On the contrary, our study found that age < =62 was an independent risk factor of distant metastasis. Also, we found that patients with distant metastatic PDAC had significantly lower levels of ALT and AST than patients without distant metastatic PDAC, which might be explained by the following reasons. First, this might be relevant to the population characteristics in our study. For example, all our patients underwent upfront surgery without neoadjuvant chemotherapy, which has liver toxicity and results in elevated levels of ALT and AST. Second, we found that patients with lower ALT levels are more likely to be without jaundice, which, on the one hand is beneficial for liver function, but on the other hand may lead to late diagnosis of PDAC due to lack of symptoms. Third, we found that patients with peritoneal metastases had a slightly lower ALT level than patients with liver metastasis (52.7 ± 139.1 vs 99.2 ± 151.3 U/L, *p* = 0.212). This implies that liver metastasis could only slightly raise the level of ALT when there are no other contributing factors.

After identifying the risk factors associated with distant metastasis, this study developed a model for predicting occult distant metastasis in patients undergoing non-curative laparotomy for potentially resectable PDAC. When a score of 6 points was taken as the cut-off value, this score system had a sensitivity of 85% and a specificity of 69%. However, it is necessary to point out that the reliability and effectiveness of this score system still needs validation by further studies. Also, because successful resection is the only cure for PDAC, these preoperative predictors alone are not contraindications for pancreatic exploration. The predictive factors identified in this study only indicated that additional preoperative staging modalities, such as selective staging laparoscopy, may be needed before laparotomy is indicated.

This study has several limitations. First, due to the nature of its retrospective design, there was a potential for several biases. For example, small intrahepatic lesions may be missed by palpation. Second, the sample size of the present study is relatively small. Therefore, a well-designed, prospective study with more data will be needed to validate the results of this study. Third, though staging laparoscopy was discussed and suggested in this study, we had limited experience in using it. Lastly, although neoadjuvant therapy has become increasingly common in the practice, our findings may not apply to this group of patients.

## Conclusions

In conclusion, we showed that for patients with potentially resectable PDAC based on MDCT, distant metastasis is still frequently encountered during surgery. Younger age, male sex, large tumor size, lower ALT and higher CA19–9 are independent predictive factors for finding distant metastasis during exploration.

## Abbreviations

ALT: Alanine aminotransferase
AST: Aspartate aminotransferase
CA19–9: Carbohydrate antigen
CEA: Carcinoembryonic antigen
DBil: Direct bilirubin
MDCT: Multidetector computed tomography
NM: No metastasis
PDAC: Pancreatic ductal adenocarcinoma
PLC: Periton eal lavage cytology
TBil: Total bilirubin
WM: With metastasis
